# Temporal Dynamics and Drivers of Ecosystem Metabolism in a Large Subtropical Shallow Lake (Lake Taihu)

**DOI:** 10.3390/ijerph120403691

**Published:** 2015-04-01

**Authors:** Zhenghua Hu, Qitao Xiao, Jinbiao Yang, Wei Xiao, Wei Wang, Shoudong Liu, Xuhui Lee

**Affiliations:** 1 Yale-NUIST Center on Atmospheric Environment, Jiangsu Key Laboratory of Agricultural Meteorology & Collaborative Innovation Center on Forecast and Evaluation of Meteorological Disasters, Nanjing University of Information Science & Technology, Nanjing 210044, China; E-Mails: qitaoxiao@163.com (Q.X.); xiaowei_522@163.com (W.X.); wangw@nuist.edu.cn (W.W.); lsd@nuist.edu.cn (S.L.); 2 Jiangsu Meteorological Bureau, Nanjing 210008, China; E-Mail: yangjb1979@gmail.com; 3 School of Forestry and Environmental Studies, Yale University, New Haven, CT 06511, USA; E-Mail: xuhui.lee@yale.edu

**Keywords:** lake metabolism, dissolved oxygen, gross primary production, ecosystem respiration, net ecosystem production, shallow lake

## Abstract

With continuous measurements of dissolved oxygen, temperature, irradiance, and wind speed, as well as frequent measurements of pH, oxidation-reduction potential, and algal chlorophyll, temporal dynamics and drivers of ecosystem metabolism in a large nutrient-rich shallow lake (Lake Taihu) are tested in this study. The results show that the dissolved oxygen concentrations in the lake fluctuate annually. They increase in autumn and winter with a peak value of 14.19 mg·L^−1^ in winter, and decrease in spring and summer with a trough value of 6.40 mg·L^−1^ in summer. Gross primary production (GPP), ecosystem respiration (R), and net ecosystem production (NEP) increase in summer, with their peak values in late summer and autumn, and decrease in winter and spring. Mean values of GPP, R and NEP are 1.75 ± 0.06 (Mean ± SE), 1.52 ± 0.05, and 0.23 ± 0.03 g O_2_ m^−3^·d^−1^, respectively. It is also found that water temperature and surface irradiance are the best predictors of GPP and R, while water temperature (wind speed) has a significantly positive (negative) relationship with NEP. The findings in this study suggest that Lake Taihu is a net autotrophic ecosystem, and water temperature and surface irradiance are the two important drivers of lake metabolism.

## 1. Introduction

The biogeochemical cycles in lake ecosystems are related to metabolic processes. Lake metabolic balances represent the difference between photosynthesis rates (CO_2_ consumption) and respiration rates (CO_2_ production) [1]. Quantifying metabolism has been an influential effort in aquatic research [2]. Gross primary production (GPP), ecosystem respiration (R), and net ecosystem production (NEP) display large temporal and spatial variations across different aquatic ecosystems [3,4,5]. NEP is useful for understanding the role of lake as sources or sinks of atmospheric CO_2_ through net heterotrophic or autotrophic annual balances, and can be used to evaluate the trophic state of ecosystems [2,5,6]. Previous studies have used measurements of GPP and R to assess the metabolic balance of aquatic ecosystems and evaluate their roles as autotrophic or heterotrophic systems [7,8]. In these studies, the average time scale is either a season or a year. However, daily measurements of these values now can be obtained by the new, reliable, and accessible technologies [9]. The continuous monitoring is not only beneficial to strengthen the predictions of how changes in environmental factors affect aquatic metabolism, but also an essential aquatic ecology method for understanding the complex changes in the biosphere [2].

The oxygen mass balance method has been applied in many previous studies of lake metabolism [3,5,10]. Measurements of diel dissolved oxygen (DO) concentrations are widely used to examine ecosystem primary production and respiration in aquatic systems, particularly in lakes [3,5,11,12,13]. Quantification and monitoring of diel DO assume that changes in DO concentration in a lake reflect the biological balance which is a result of photosynthetic production and respiratory consumption as well as physical exchange of oxygen between air and water. The diel DO method has advantages such as no bottle and container effects, ease of data collection and measurement of whole-ecosystem metabolism, and the potential of providing estimate of spatial and temporal variability [14]. Therefore, the long-term monitoring of DO dynamics in lakes plays a significant role in quantification of lake metabolism.

Previously, autotrophy or metabolic balance has been assumed to occur in most aquatic systems [15]. However, it is now proven that the majority of freshwater systems are heterotrophic [16,17,18]. Metabolism is driven by different physical, chemical, and biological forces, such as temperature, irradiance, wind speed, pH, dissolved organic carbon (DOC), inorganic nitrogen, total phosphorus (TP), chlorophyll-*a* (Chl *a*), and phytoplankton, *etc.* [3,18,19,20,21]. Although recently there have been numerous advances in the study of ecosystem metabolism [2,22], the majority of this research has been conducted in temperate waters, with little in subtropical and tropical waters [2], and even fewer studies examine eutrophic subtropical shallow lakes, where sunlight can reach the benthal layer and water column productivity is high [23].

Lake Taihu is a eutrophic subtropical lake, and the third largest freshwater lake in China. The watershed is located in the Yangtze River Delta and is the most densely populated (about 1100 inhabitants km^−2^) and economically developed area in China (contributing about 13% of the nation’s gross domestic product). Since the 1980s, the lake has become eutrophic due to its multiple uses [24]. Total nitrogen (TN) ranged between 0.38 and 21.93 mg·L^−1^, TP varied from 0.013 to 1.459 mg·L^−1^, DOC had an annual mean concentration of 3.76–8.97 mg·L^−1^, and Chl *a* concentration reached a peak of 327.6 μg·L^−1^ [25]. Lake Taihu, with high production in the littoral zone, has simultaneously high nutrient and DOC concentrations, and will fall on the high end of both primary productivity and DOC gradients, while the metabolic balance remains uncertain.

Lake Taihu can serve as a representative of other eutrophic shallow lakes worldwide. In this study, diel monitoring of DO in the lake is conducted to estimate its primary production and ecosystem respiration. Then, temporal patterns of DO concentrations and lake metabolic rates are presented based on 2-year DO measurements. Hypothetically, there are high nutrient concentrations, dissolved organic matter, and sufficient light intensity in this shallow lake, and phytoplankton and macrophyte will have high GPP and biomass, which lead to low net heterotrophy in Lake Taihu. The objectives of this study are to justify the hypotheses, and explore fundamental questions regarding the metabolism of this large shallow eutrophic lake. Specifically, seasonal variability in the metabolism rates will be characterized, the question that whether the lake is a net autotrophic or a net heterotrophic lake will be answered, and the important physical and biological drivers involved will be identified.

## 2. Experimental Section

### 2.1. Site Description

**Figure 1:**
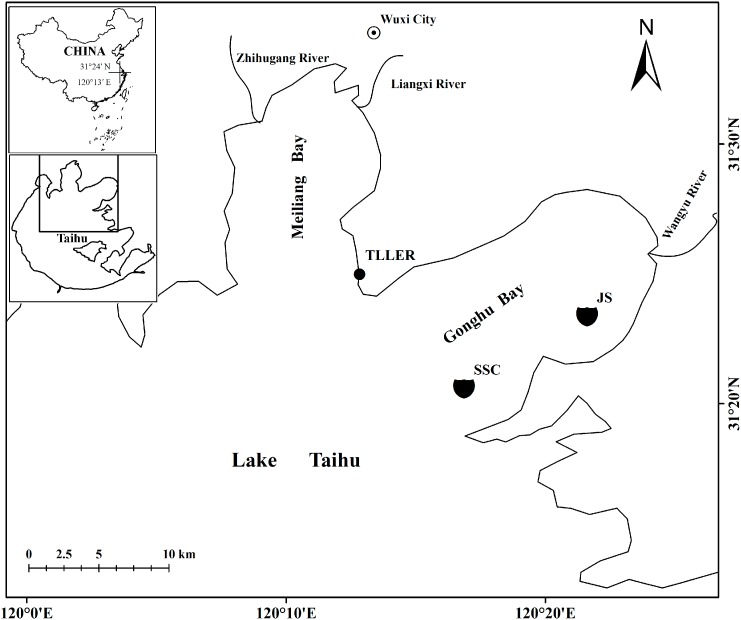
The map of the study area, and the location of the two sampling sites—Shangshancun (SSC) and Jinshu (JS) buoy stations. The downwelling shortwave radiation was collected at the Taihu Laboratory for Lake Ecosystem Research (TLLER).

The experiment was conducted in Lake Taihu (Figure 1), from November 2008 to November 2010. Lake Taihu (30°05′–32°08′ N, 119°08′–121°55′ E) is a naturally large shallow eutrophic lake located in the delta of the Yangtze River in Eastern China. The lake has a surface area of 2.4 × 10^3^ km^2^, a catchment area of 3.65 × 10^4^ km^2^, a maximum depth of 2.6 m, and a mean depth of 1.9 m [24]. Lake Taihu has negligible vertical thermal stratification gradients because of its shallowness [26].

Two buoy stations were moored in Gonghu Bay, northeastern Lake Taihu (Figure 1), which is an important drinking water source supplying for Wuxi City. To improve water quality, water is transferred from the Yangtze River to the lake through Wangyu River. The average depth of Gonghu Bay is 2.0 m, and the surface area is 147 km^2^, half of which is covered by abundant macroplant vegetation [27]. The main species of Shangshancun station (SSC, 31°21′ N, 120°17′ E) are *Potamogeton malaianus* and *Myriophyllum spicatum*. There are lots of species in Jinshu station (JS, 31°23′ N, 120°22′ E), including *P. maackianus*, *P. malaianus*, *M. spicatum*, *Elodea nuttallii*, *Vallisneria natans*, *Ceratophyllum demersum*, *Nymphoides peltata*, and *Trapa natans*.

The downwelling shortwave radiation was collected at the Taihu Laboratory for Lake Ecosystem Research (TLLER, 31°24′ N, 120°13′ E), Nanjing Institute of Geography and Limnology, Chinese Academy of Sciences, lie in Meiliang Bay, about 8.7 km linear distance from the SSC buoy station, from June to November in 2010. Meiliang Bay is one of the most eutrophic bays in Lake Taihu, where two rivers (Liangxi and Zhihugang Rivers) empty large amounts of wastewater from factories and residential areas into the bay [28].

### 2.2. In Situ Measurements

Continuous measurements of some physical, chemical, and biological parameters were performed at two moored buoys (JS and SSC). Parameters including water temperature, turbidity (Turb), pH, DO, oxidation-reduction potential (ORP), and Chl *a* were measured using a continuously recording multiparameter underwater sensor (YSI 6600, Yellow Springs Instruments, Yellow Springs, OH, USA). These parameters were collected at 2-h intervals from 28 October 2008 to 10 November 2010 in SSC station and from 30 July 2010 to 10 November 2010 in JS station. Interruption of measurements occasionally occurred for the malfunction of instruments. The probes were hung from the buoy and took measurements sequentially at 0.5, 1, and 1.5 m depth. The DO probes were first calibrated in air-saturated water prior to deployment, while after that they were calibrated in air-saturated water weekly. The monitoring accuracy of water temperature, Turb, pH, DO, ORP, and Chl *a* are 0.15 °C, 0.3 NTU, 0.2, 0.1 mg·L^−1^, 20 mV, and 0.1 μg·L^−1^, respectively. In addition, a HOBO H21 micro meteorological station (Onset Computer Corporation, Bourne, MA, USA) was mounted on each of the buoys. The following meteorological data were collected at 5-min intervals and recorded as one hour averages: Air temperature, relative humidity, wind speed, and wind direction. The monitoring accuracy of these parameters are 0.2 °C, 2.5%, 0.5 m·s^−1^, and 5°, respectively.

### 2.3. Quantification of Lake Metabolism

To estimate the lake metabolism, DO mass balance model is used here. It includes the following equations [12]:

∆O_2_/∆*t* = GPP – R – *F*/*Z*_mix_ – *A* (1)

where ∆O_2_/∆*t* is the change in DO concentration over time (mg O_2_·L^−1^·h^−1^), GPP is the primary production (g O_2_·m^−3^·h^−1^), R is the ecosystem respiration (g O_2_·m^−3^·h^−1^), *F* is the oxygen exchange with the atmosphere (g O_2_·m^−2^·h^−1^), it is negative (positive) for downward (upward) flux of O_2_. *Z*_mix_ (m) is the mixed layer depth (m). Since Lake Taihu is a shallow lake and is not subject to thermal stratification, *Z*_mix_ is assumed equal to the mean depth of 1.9 m. The term *A* includes all the other processes that change DO concentration, such as horizontal and vertical advection within the lake. For simplicity, it is omitted in this study.

Following Staehr *et al.* [12], the oxygen exchange with the atmosphere (*F*) is computed as:

*F* (g O_2_ m^−2^ h^−1^) = *k* (O_2 meas_ – O_2sat_) (2)

where O_2 meas_ is the actual DO concentration (mg·L^−1^); O_2sat_ is the oxygen saturation (mg·L^−1^) as a function of water temperature (T, kelvin). The equation of Weiss [29] which is used to calculate O_2sat_ has been corrected for altitude according to the United States Geological Survey (USGS) Water Quality Technical Memoranda 81.11 and 81.15 [30]. The coefficient *k* (m·h^−1^) is estimated using the Schmidt coefficient (*Sc*) and gas piston velocity corresponding to a Schmidt coefficient of 600 (*k*_600_):

*k* (m·h^−1^) = *k*_600_ × (*Sc*/600)^−n^ (3)

where *n* is equal to 2/3 for wind speed at 10 m height (U_10_) < 3.7 m·s^−1^ and is equal to 0.5 for U_10_ > 3.7 m·s^−1^. The Schmidt coefficient is calculated from water temperature (T, Celsius) using the equation of Wanninkhof [31]:

*Sc* = 1800.6 – 120.1 T + 3.7818 T^2^ – 0.0476 T^3^ (4)

The coefficient *k*_600_ (m·h^−1^) depends on wind speed at 10 m height and is calculated using the equation of Cole and Caraco [32]:

*k*_600_ (m·h^−1^) = (2.07 + 0.215 U_10_^1.7^)/100 (5)

U_10_ is calculated according to Smith [33]. Other equations from Staehr *et al.* [12] which are used to calculate metabolism are the following:

NEP_hr_ (g O_2_·m^−3^·h^−1^) = ∆O_2_ – *F*/*Z*_mix_ (6)

NEP_daytime_ (g O_2_·m^−3^·daylight period^−1^) = mean NEP_hr_ during daylight × day fraction × 24 (7)

R_hr_ (g O_2_·m^−3^·h^−1^) = mean NEP_hr_ during darkness (8)

R_daytime_ (g O_2_·m^−3^·daylight period^−1^) = R_hr_ × 24 h × dayfraction (9)

R_day_ (g O_2_·m^−3^·d^−1^) = R_hr_ × 24 h (10)

GPP (g O_2_·m^−3^ d^−1^) = NEP_daytime_ + R_daytime_ (11)

NEP (g O_2_·m^−3^·d^−1^) = GPP – R_day_ (12)

where NEP_daytime_ is the daytime net ecosystem production; NEP_hr_ is the hourly net ecosystem production; R_hr_ is the mean nighttime ecosystem respiration; R_daytime_ is the daytime ecosystem respiration; R_day_ is the total ecosystem respiration; dayfraction = light hours/24 h, and light hours is determined from day of year (DOY) and latitude, and the calculation procedure can be found in Staehr *et al.* [12].

### 2.4. Statistical Analyses

Pearson correlation analysis are used to evaluate correlations between metabolic rates and physical, chemical, and biological drivers. Significance referred to in the text is at the *p* = 0.05 level unless otherwise stated. All data analyses are performed using SPSS 13.0 (SPSS Inc., Chicago, IL, USA).

## 3. Results

### 3.1. Temporal Pattern in DO Concentrations

#### 3.1.1. Temporal Dynamics of DO Concentration

**Figure 2:**
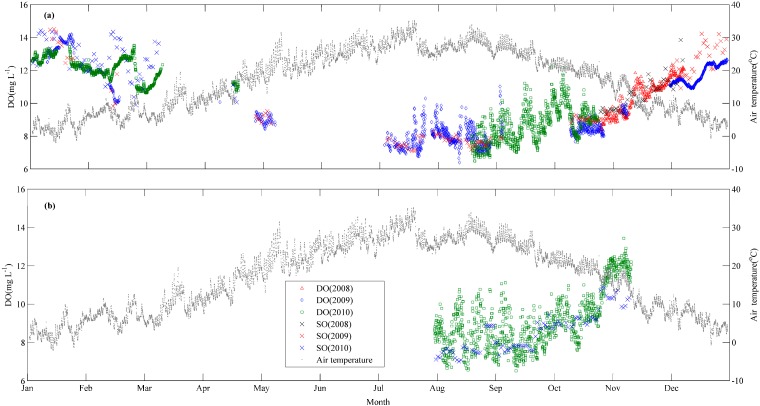
Temporal variation of dissolved oxygen (DO) and saturated oxygen (SO) concentrations at (**a**) Shangshancun (SSC) buoy station and (**b**) Jinshu (JS) buoy station.

Figure 2 shows the temporal dynamics of DO. The DO concentrations in three years (2008, 2009, and 2010) show similar seasonal patterns. They increase with time during autumn and winter, reaching a maximum value of 14.19 mg·L^−1^ in winter, and then decline in spring and summer, reaching a minimum value of 6.40 mg·L^−1^ in the summer. The median value of DO was 9.78 mg·L^−1^.

The DO concentration changed with temperature and biological activities. Driven by seasonal temperature changes, the saturation DO had a significant seasonal pattern, with lower values in summer and higher values in winter, and with the opposite tendency with respect to temperature. The saturation DO values were higher than the observed DO concentration in winter, while the observed DO concentration was higher than the saturation DO value during summer and autumn.

#### 3.1.2. Seasonal and Diurnal Changes of DO

**Figure 3:**
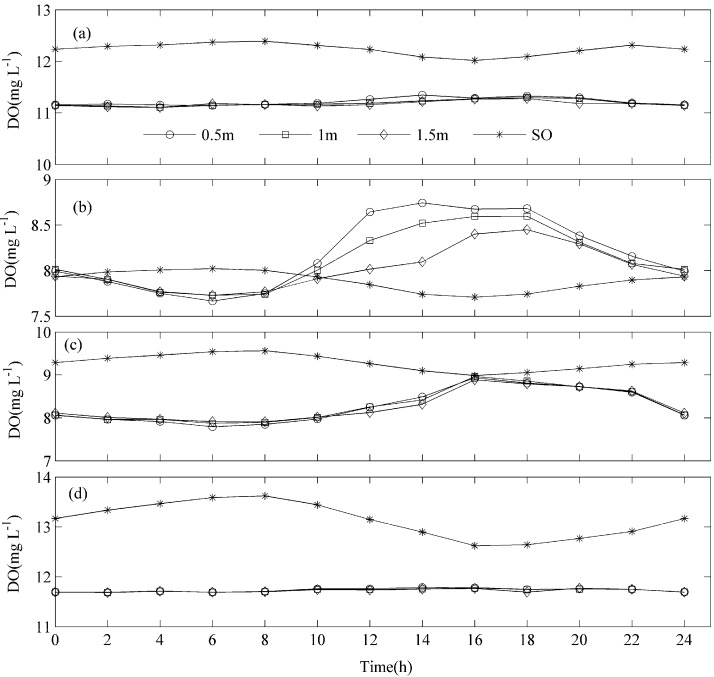
Seasonal changes in saturated oxygen (SO) and dissolved oxygen (DO) concentrations at different depths. (**a**), (**b**), (**c**), and (**d**) represent the variation in spring, summer, autumn, and winter, respectively.

The diurnal average DO concentrations at the 50-cm depth were 11.22 ± 0.02 (Mean ± SE), 8.20 ± 0.12, 8.28 ± 0.12, and 11.73 ± 0.01 mg·L^−1^ during spring, summer, autumn, and winter, respectively. The highest 2-h mean DO concentration was 11.79 mg·L^−1^ and occurred in winter, while the lowest DO concentration of 7.67 mg·L^−1^ was recorded in summer (Figure 3). Large diurnal variations of DO concentrations were observed in summer and autumn, which may be caused mainly by photosynthesis of aquatic plants and phytoplankton. In these seasons the highest DO concentration appeared between 16:00 and 18:00 local time, and the lowest at 06:00 local time. The DO increased gradually from 06:00, reached the highest maximum value around 16:00 to 18:00, and then decreased gradually with time.

The DO concentrations at the 50, 100, and 150 cm depths show similar diurnal changes patterns for different seasons (Figure 3). In summer, significant vertical DO patterns in depth were observed in the daytime from 10:00 to 18:00, which may be caused by photosynthesis of aquatic plants and phytoplankton.

### 3.2. Temporal Pattern in Lake Metabolism

#### 3.2.1. Temporal Dynamics of Metabolism Rates

**Figure 4:**
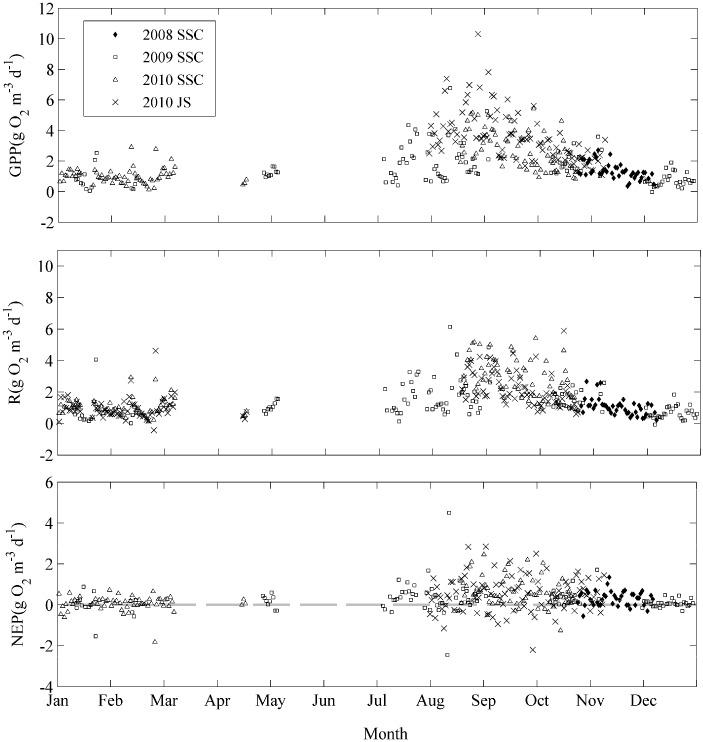
The temporal dynamics of gross primary production (GPP), ecosystem respiration (R), and net ecosystem production (NEP) rates at Shangshancun (SSC) and Jinshu (JS) buoy stations.

**Figure 5:**
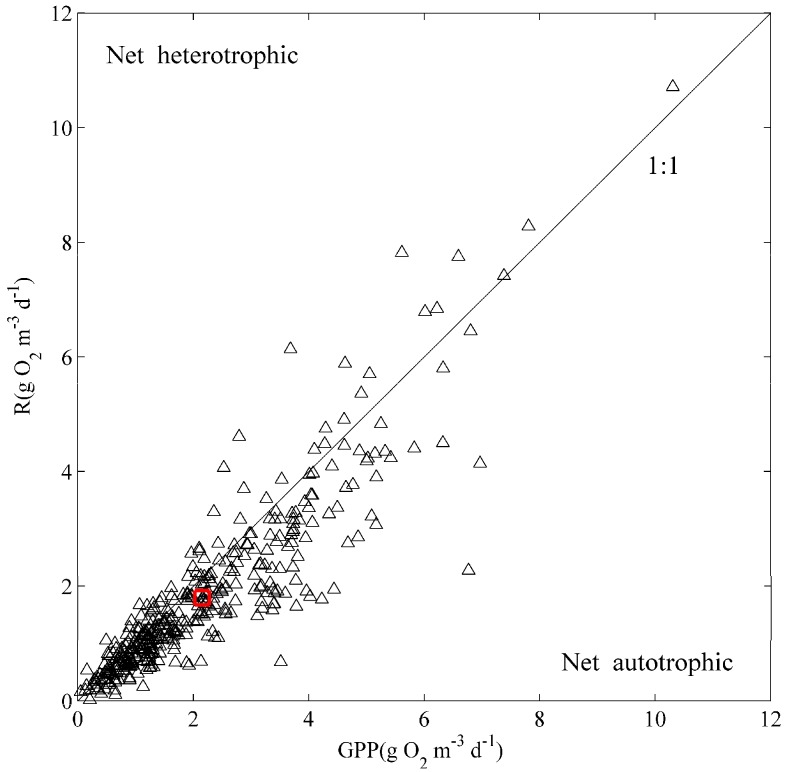
The relationship between gross primary production (GPP) and ecosystem respiration (R). Days that fall above the 1:1 line are net heterotrophic and days that fall below the 1:1 line are net autotrophic. The red circular symbol shows the mean value.

GPP, R, and NEP show similar seasonal change patterns (see Figure 4). The rates of GPP, R, and NEP reach their maximum values in the late summer and kept decreasing in winter and spring. The annual mean rates of GPP, and R were 1.75 ± 0.06, and 1.52 ± 0.05 g O_2_·m^−3^·d^−1^, respectively. GPP:R ratio is 1.15 (Figure 5). The lake was net autotrophic with an annual average NEP 0.23 ± 0.03 g O_2_·m^−3^·d^−1^. Net heterotrophy occurred in all the months, but it was more frequent during the autumn and winter when the NEP values were significantly below 0.

#### 3.2.2. Seasonal Changes of Metabolism Rates

**Figure 6:**
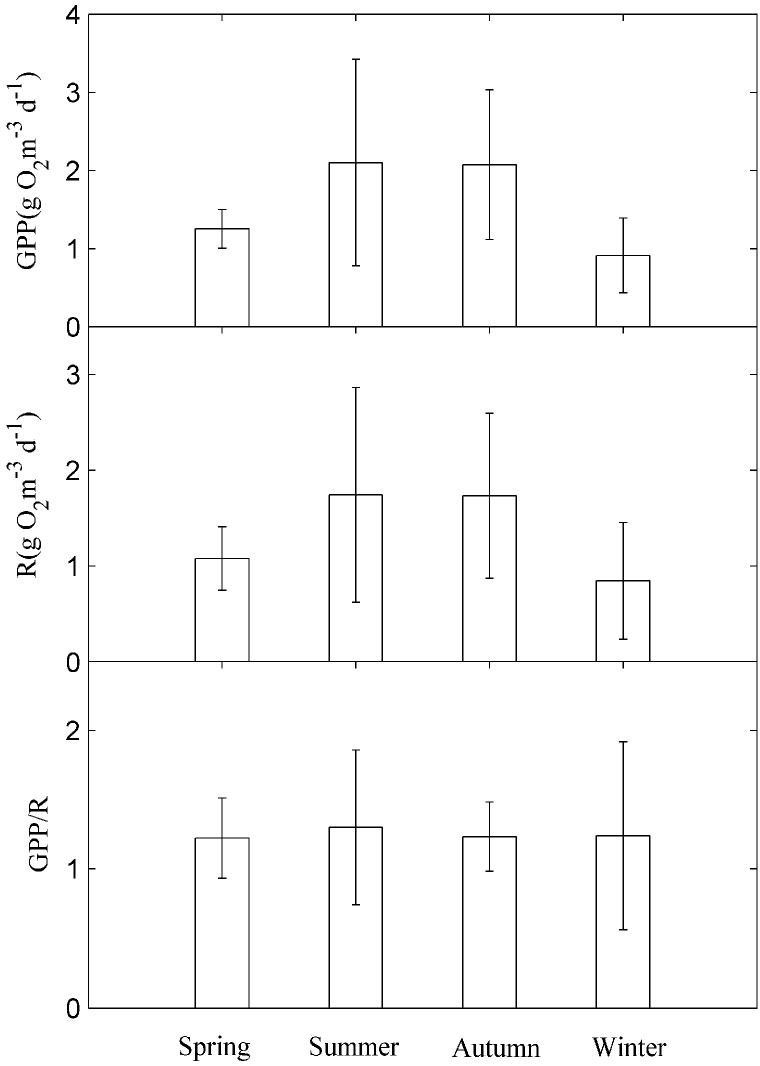
Seasonal values in gross primary production (GPP), ecosystem respiration (R), and GPP/R. Data are presented as the mean values and the error bars are SDs.

There were higher (smaller) GPP, and R rates in summer and autumn (winter) (Figure 6). The average rates of GPP and R were 2.10 ± 1.32, 1.74 ± 1.12 g O_2_·m^−3^·d^−1^ in the summer, and 2.07 ± 0.96 and 1.73 ± 0.86 g O_2_·m^−3^·d^−1^ in the autumn, respectively.

The average GPP and R rates were 0.91 ± 0.48, 0.85 ± 0.61 g O_2_·m^−3^·d^−1^ in the winter, respectively. The mean GPP/R values were 1.22 ± 0.29, 1.30 ± 0.56, 1.23 ± 0.25, and 1.24 ± 0.68 during the spring, summer, autumn, and winter, respectively.

### 3.3. Drivers of Lake Metabolism

**Figure 7:**
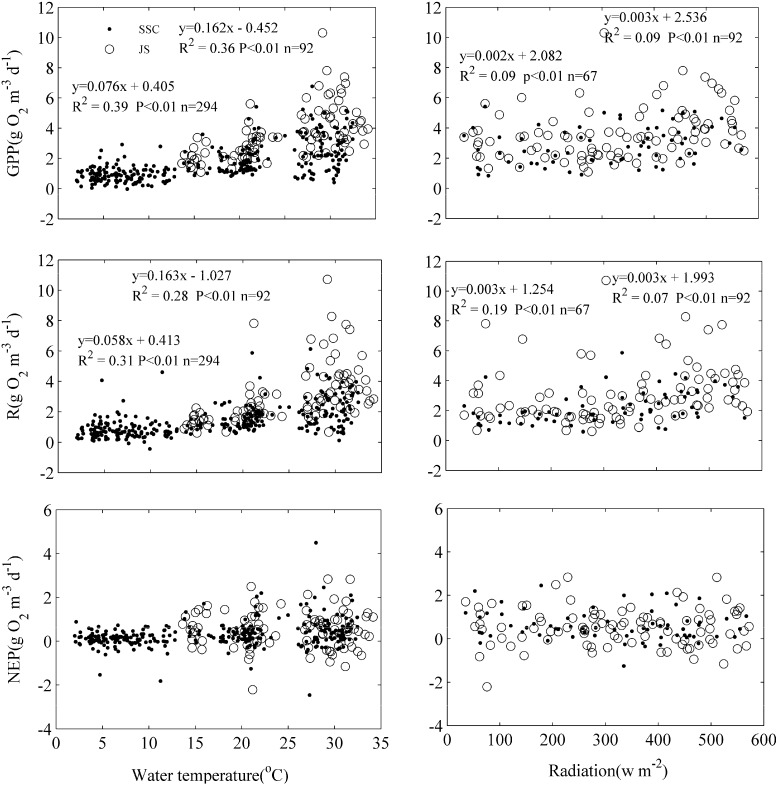
Relationships between daily values of gross primary production (GPP), ecosystem respiration (R), net ecosystem production (NEP), daily average values of water temperature, and surface radiation.

GPP and R rates were strongly stimulated by increasing water temperature and surface irradiance (Figure 7; Table 1), explaining the contrasts between high rates from June to September and low rates outside this period.

**Table 1:** Relationships between metabolism and environmental factors in Shangshancun (SSC) and Jinshu (JS) buoy stations.

		T_w_ (°C)	Radiation (w·m^−2^)	Wind Speed (m·s^−1^)	pH	ORP (mV)	Chl *a* (μg·L^−1^)	Turb (NTU)
SSC (*n* = 294)	GPP (g O_2_·m^−3^·d^−1^)	0.622 **	0.269 *	−0.132 *	−0.133 *	−0.360 **	0.032	0.112
	R (g O_2_·m^−3^·d^−1^)	0.553 **	0.430 **	−0.081	−0.089	−0.339 **	0.022	0.122 *
	NEP (g O_2_·m^−3^·d^−1^)	0.294 **	– 0.149	−0.125 *	−0.113	−0.139	0.026	0.014
JS (*n* = 92)	GPP (g O_2_·m^−3^·d^−1^)	0.597 **	0.299 **	−0.187	−0.235 *	−0.180	0.522 **	−0.210
	R (g O_2_·m^−3^·d^−1^)	0.529 **	0.263 *	−0.241 *	−0.217 *	−0.169	0.426 **	−0.196
	NEP (g O_2_·m^−3^·d^−1^)	−0.010	−0.001	0.169	0.023	0.022	0.071	0.029

*, ** Correlation is significant at the 0.05, and 0.01 level (2-tailed), respectively. GPP, gross primary production; R, ecosystem respiration; NEP, net ecosystem production; Tw, water temperature; ORP, Oxidation reduction potential; Chl *a*, chlorophyll-*a* concentration; Turb, turbidity.

The relative importance of physical and biological drivers for GPP, R and NEP is shown in Table 1. It can be seen that temperature and surface irradiance are the best predictors of GPP and R. Wind speed, pH, and ORP are negatively related to GPP, R and NEP rates at the SSC station (Table 1). Chl *a* is positively related to GPP and R rates at the JS station. NEP has a significant positive relation with temperature and a negative relation with wind speed at the SSC station.

## 4. Discussion

### 4.1. Seasonal Patterns of Lake Metabolism

Calculations of lake metabolism from continuous daily oxygen measurements show large variations in GPP, R, and NEP across seasons. GPP and R rates display similarly high levels in summer and low values in winter in the present study, which is consistent with previous studies [5,34]. The seasonal patterns of GPP and R rates are in accordance with water temperature and surface radiation. In present study, there is a positive relationship between GPP and R rates, which is also in agreement with previous studies on lake metabolism [3,5].

Estimates of daily GPP and R are based on the classical assumption that R_night_ and R_light_ are equal. However, some previous studies suggest that R_light_ tends to be equal to or larger than R_night_ as a result of involvement of several biochemical pathways in addition to the mitochondrial dark respiration [5,35,36]. Because no method is available to measure R_light_ on a routine and continuous basis, uncertainty in the estimates of GPP and R cannot be avoided in this study. Moreover, if R_light_ exceeds R_night_, the magnitude of GPP and R would be increased by equal amounts, the influence on the GPP:R ratio would remain small so that NEP does not change [3,5].

### 4.2. Net Autotrophic Lake

The amount of organic matter produced and consumed within a lake determines its net metabolic balance. Aquatic systems where GPP exceeds R have positive NEP and are net autotrophic, whereas aquatic systems where more organic matter is respired than produced have negative NEP and are net heterotrophic [22]. If autotrophic, the ecosystem will produce more organic carbon and export or accumulate organic carbon. On the contrary, if heterotrophic, the ecosystem will be dependent on organic matter inputs from adjacent ecosystems [37].

Some previous studies suggest that aquatic ecosystems are frequently net heterotrophic, with respiration exceeding primary production, indicating that the food web of the ecosystem is supported by external inputs of energy [16,18,19,38]. In present study, it is found that annual mean GPP:R ratio is 1.15, indicating that Lake Taihu acts as a net autotrophic lake (Figure 5). Lake Taihu was hypothesized to have net autotrophy because increasing net autotrophic tends to occur in nutrient-rich and productive aquatic ecosystems [19,39,40]. Previous studies have also shown that a net heterotrophic metabolism is most likely in unproductive ecosystem, and highly productive ecosystems tend to be net autotrophic [18].

Liu *et al.* found that Lake Taihu was net heterotrophic [23]. They indicated that the high levels of suspended solids and nutrient concentrations limited underwater light which reduced phytoplankton photosynthesis, and the high temperature was good for plankton repiration [23]. The study site of Liu *et al.* is situated in Meiliang Bay, in northern Lake Taihu (Figure 1), which is one of the most eutrophic bays. Two main rivers (Liangxi River and Zhihugang River) empty large amounts of wastewater from factories and residential areas into the bay [28]. The high nutrient level and temperature make Meiliang Bay display net heterotrophy [23]. In the present study, two buoy stations (SSC and JS) are situated in Gonghu Bay, in northeastern Lake Taihu (also see Figure 1), where half of its area covered by abundant macroplant vegetation [27]. Gonghu Bay is an important source of drinking water for Wuxi City and part of Suzhou City. To improve water quality, water transfers from the Yangtze River to the lake through Wangyu River. Hu *et al.* found that the Yangtze River water transfer have notable positive effects on water quality in Gonghu Bay [27]. Moreover, Gonghu Bay is a submerged macrophyte habitat, flourishing from early April to late October every year [41]. Gonghu Bay is mesotrophic, while Meiliang Bay is hypertrophic. The annual mean underwater transparency of Gonghu Bay and Meiliang Bay are 0.58 m and 0.26 m, respectively. There is enough underwater light for aquatic plants’ photosynthesis in Gonghu Bay, while, underwater light for photosynthesis is lacking in Meiliang Bay. In the eutrophic Meiliang Bay, GPP:R ratios are usually less than 1.0, except for several occasions, such as February and April when ratios were slightly above 1.0 [23]. Menawhile, in the mesotrophic Gonghu Bay, the average GPP:R ratios during spring, summer, autumn, and winter are 1.22, 1.30, 1.23, and 1.24, respectively, showing a net autotrophic metabolism.

In present study, GPP exceeded R at the GPP rate above 82 μmoL O_2_·L^−1^·d^−1^, which matches well with Cole *et al.*, who found that lakes on an average were net autotrophic at GPP values above 80 μmoL O_2_·L^−1^·d^−1^ [3]. Staehr and Sand-Jensen found that Lake Frederiksborg Slotssø located on Zealand was net autotrophic when the GPP was about 90 μmol O_2_·L^−1^·d^−1^ [5].

### 4.3. Drivers of Lake Ecosystem Metabolism

GPP and R rates are not only controlled by environmental and biological factors which determine the biomass and physiological state of lake phytoplankton, but also by factors which determine the daily production rates for a certain biomass and species composition [5]. In present study, GPP and R rates were strongly positively related to water temperature and irradiance. Specifically, water temperature was the strongest factor when the entire data was analyzed (Table 1). These results are in agreement with Staehr and Sand-Jensen [5]. The apparent temperature effect also was coupled with available irradiance. High summer temperature and irradiance were associated with formation of blue-green algal blooms and consequently high rates of GPP and R, making the lake net autotrophic during the summer.

In present study, a negative relationship between GPP and wind speed in both buoy stations is found (Table 1). It is because that high wind speed accelerates deeper vertical mixing and lower light availability in the water column, resulting in the reduction of GPP. A positive relationship is found between R and turbidity at the SSC station, which is consistent with the result of DeNicola *et al.* [42]. This maybe explained, in part, by the relationship between turbidity and phytoplankton concentrations.

Over longer timescales of weeks and months, the biomass and physiological state of phytoplankton are markedly influenced by inorganic nutrient availability, mixing regime and food web structure in addition to the impact of temperature and irradiance [43]. In accordance with previous studies [5], high pH and ORP can also constrain photosynthetic production (Table 1), because more dissolved inorganic carbon is changed into CaCO_3_ that precipitates at high pH and ORP.

There are similar dynamic metabolism patterns between the two buoy stations (JS and SSC), which are net autotrophic. From August to October, the maximum GPP values of JS and SSC stations are 10.31 and 6.77 g O_2_·m^−3^·d^−1^, mean GPP rates of JS station and SSC station are 3.66 ± 1.67 and 2.52 ± 1.28 g O_2_·m^−3^·d^−1^, respectively, while no significant differences in GPP, R, and NEP between the JS station and SSC station (*p* = 0.183, 0.437, and 0.355) were found. Possible explanations for the differences of GPP rates at the two buoy stations include: (1) there are more abundant macrophytes (species and biomass) at the JS station, such as *P. maackianus*, *P. malaianus*, *M. spicatum*, *Elodea nuttallii*, *Vallisneria natans*, *Ceratophyllum demersum*, *Nymphoides peltata*, and *Trapa natans*. (2) Compared to the JS station, the site of the SSC station is close to the main lake, where underwater light may be limited because of the higher turbidity, resulting in an adverse effect on macrophyte photosynthesis.

## 5. Conclusions

With the data of dissolved oxygen, temperature, irradiance, and wind speed, the temporal dynamics and drivers of ecosystem metabolism in Lake Taihu are analyzed. It is noted that dissolved oxygen concentrations kept increasing (decreasing) during autumn and winter (spring and summer) with its maximum (minimum) value showing in winter (summer). Gross primary production, ecosystem respiration, and net ecosystem production kept increasing (decreasing) during summer and autumn (winter and spring) with a maximum value in late summer. The results also showed that Lake Taihu is a net autotrophic ecosystem, and water temperature and surface irradiance are two important drivers of the lake metabolism.
